# Prognostic Impact of Modified H2FPEF Score in Patients Receiving Trans-Catheter Aortic Valve Replacement

**DOI:** 10.3390/jcm12165396

**Published:** 2023-08-19

**Authors:** Kousuke Akao, Teruhiko Imamura, Shuhei Tanaka, Hiroshi Onoda, Ryuichi Ushijima, Mitsuo Sobajima, Nobuyuki Fukuda, Hiroshi Ueno, Koichiro Kinugawa

**Affiliations:** The Second Department of Internal Medicine, University of Toyama, Toyama 930-0194, Japan; apollon080607@gmail.com (K.A.); honoda@med.u-toyama.ac.jp (H.O.);

**Keywords:** heart failure, hemodynamics, diastolic dysfunction, valvular disease, aortic valve disease

## Abstract

Background: H2FPEF is a recently introduced score for the diagnosis of heart failure with preserved ejection fraction (HFpEF). Many patients with severe aortic stenosis have clinical/subclinical HFpEF and have worsening heart failure even after trans-catheter aortic valve replacement (TAVR). We investigated the prognostic impact of the H2FPEF score in TAVR candidates. Methods: Patients undergoing TAVR procedures at a single academic center between 2015 and 2022 were included. The H2FPEF score was calculated using baseline characteristics before TAVR. The prognostic impact of the score on the post-TAVR composite endpoint, consisting of all-cause death and heart failure readmissions during the 2-year observation period, was evaluated. Results: A total of 244 patients (median age 86 years, 70 males) were included. The median value of H2FPEF score was 3 (2, 4). The score was significantly associated with the primary outcome with a hazard ratio of 1.33 (95% confidence interval 1.02–1.74, *p* = 0.036). We constructed a modified H2FPEF score by adjusting cutoffs of several items for better prognostic stratification (i.e., age and body mass index). A modified score had a higher area under the curve than the original one (0.65 vs. 0.59, *p* = 0.028) and was independently associated with the primary outcome with an adjusted hazard ratio of 1.22 (95% confidence interval 1.01–1.49, *p* = 0.047). Conclusions: A modified H2FPEF score, which was originally developed to diagnose the presence of HFpEF, could be used to risk-stratify elderly patients receiving TAVR. The clinical utility of this score should be validated in future studies.

## 1. Background

Trans-catheter aortic valve replacement (TAVR) was introduced as a less invasive intervention for severe aortic stenosis, initially in patients at high risk for surgical valve replacement [1,2] and currently in patients with less sick cohorts [3], as endorsed by the guidelines [4,5]. The clinical outcomes after TAVR have further improved due to improvements in sedation technique, smaller sheaths for vascular access, innovation of vascular closure devices, and more sophisticated peri-procedural management [6,7,8]. 

Nevertheless, heart failure recurrence after TAVR is one of the unsolved issues [9]. Many patients with severe aortic stenosis have heart failure with preserved ejection fraction (HFpEF) with left ventricular hypertrophy, impaired diastolic function due to a long-standing increase in afterload, and lower stroke volume [10,11,12]. Patients with reduced ejection fraction due to severe aortic stenosis also have diastolic dysfunction [13,14]. Such an impaired cardiac function persists even after TAVR [15]. However, the detailed association between pre-TAVR degree of diastolic dysfunction and clinical outcomes after TAVR remains unknown. 

The diagnosis of HFpEF is sometimes challenging. Several scores have been introduced to help us diagnose HFpEF, such as H2FPEF score [16]. The H2FPEF score consists of several easily available clinical parameters, including body mass index, the presence of hypertension, the presence of atrial fibrillation, pulmonary hypertension, age, and echocardiographic E/e’ ratio. H2FPEF score may be useful not only for the diagnosis of HFpEF but also for risk-stratifying heart failure patients [17]. We hypothesized that the H2FPEF score would be associated with clinical outcomes after TAVR. In this study, we evaluated the prognostic impact of H2FPEF score in patients receiving TAVR. We further modified the score to improve its predictability in the contemporary TAVR candidates who were elderly with advanced sarcopenia. 

## 2. Methods

### 2.1. Patient Selection

Consecutive patients with severe aortic stenosis undergoing TAVR procedures at a large academic center, University of Toyama, between 2015 and 2022 were prospectively included in the institutional registry database, and this study was retrospectively conducted using this database. All patients were followed for 2 years or until May 2023 unless lost to follow-up. H2FPEF score, which was originally introduced to diagnose HFpEF [16], was calculated using baseline characteristics before TAVR. Patients with missing data were excluded. Written informed consents were obtained from all participants on admission. The institutional review board approved the study protocol. 

### 2.2. Calculation of H2FPEF Score

H2FPEF score was calculated in all participants using baseline characteristics before TAVR, by assigning each a weighted score if patients satisfied the variables, including body mass index >30 (2 points), use of multiple anti-hypertension medications (1 point), atrial fibrillation (both persistent and paroxysmal) (3 points), pulmonary hypertension with estimated pulmonary artery systolic pressure by echocardiography >35 mmHg (1 point), age >60 years (1 point), and Doppler echocardiographic E/e’ ratio >9.0 (1 point) [16]. The total score was calculated as a summation of these scores, ranging between 0 and 9 points.

We further constructed a modified H2FPEF score in this study by updating cutoffs of body mass index and age, because most TAVR candidates had advanced sarcopenia and probably did not satisfy body mass index >30. Also, most TAVR candidates were elderly aged over 60 years. We believed that the original H2FPEF score should be updated to better fit the contemporary TAVR candidates. 

### 2.3. Other Baseline Characteristics

Baseline demographics, laboratory, echocardiographic, and medication data before TAVR were obtained as baseline characteristics. 

### 2.4. TAVR Procedure

Patients with symptomatic severe aortic stenosis with peak velocity >4.0 m/s, mean pressure gradient >40 mmHg, or aortic valve area <1.0 cm^2^ were eligible for TAVR. The indication for TAVR was determined by the heart valve team conference consisting of cardiac surgeons, interventional cardiologists, anesthesiologists, nurses, and imaging specialists. Patients underwent standard TAVR procedure using the Edwards Sapien XT/Sapien 3 Transcatheter Heart Valve (Edwards Lifesciences, Irvine, CA, USA) or the Medtronic CoreValve/Evolut R Revolving System (Medtronic, Minneapolis, MN, USA). An antithrombotic regimen following TAVR was used at the discretion of the attending physician. 

### 2.5. Post-TAVR Course and Primary Outcome

Patients were followed at our center or affiliated centers by board-certified cardiologists every 1–2 month(s) at out-patient clinic in a standard manner. Day 0 was defined as the time of TAVR procedure. The observation period was 2 years or until May 2023 from day 0. Clinical outcomes including death and heart failure readmissions were counted. The primary outcome was a composite of all-cause death and heart failure readmissions. 

### 2.6. Statistical Analysis

Continuous variables were presented as median and interquartile range and compared using Mann–Whitney U test. Categorical variables were presented as numbers and percentages and were compared using Fisher’s exact test. A value of 2-tailed *p* < 0.05 was considered statistically significant. Statistical analyses were performed using SPSS Statistics 23 (SPSS Inc., Armonk, IL, USA).

An independent variable was the H2FPEF score, which was modified as detailed below. Patients were followed for 2 years or until May 2023 from the TAVR procedure (day 0). The primary outcome was all-cause death and heart failure readmissions. 

Cox proportional hazard ratio regression analyses were performed to evaluate the prognostic impact of the H2FPEF score (and the modified H2FPEF score). Potential confounders were considered to be included in the multivariable analyses for the adjustment after confirmation of statistical significance in the univariable analyses, including age, male sex, body mass index, serum albumin, estimated glomerular filtration ratio, plasma B-type natriuretic peptide, left ventricular ejection fraction, heart failure history, and atrial fibrillation. Receiver operating characteristics analyses were performed to evaluate the prognostic impact and calculate cutoffs of variables for the primary outcome. Kaplan–Meier analysis with log-rank test was performed for risk stratification using a modified H2FPEF score. 

H2FPEF score was modified by adjusting cutoffs of body mass index and age, which were calculated using receiver operating characteristics analyses, for better adjusting in the current TAVR candidates (i.e., elderly patients with advanced sarcopenia). 

## 3. Results

### 3.1. Baseline Characteristics

A total of 352 patients were eligible for this study. Of them, 108 with missing data were excluded. Finally, 244 patients (median 86 years, 70 males) were included (Table 1). Median body mass index was 21.5 (19.1, 24.3). There were 178 patients (73%) with hypertension and 25 patients (10%) had atrial fibrillation (persistent or paroxysmal). Peak velocity at aortic valve was 4.4 (4.0, 4.9) m/s on median. Right ventricular systolic pressure was 31 (26, 37) mmHg on median. E/e’ ratio was 16.3 (12.4, 23.1) on median. 

### 3.2. H2FPEF Score Calculation

**Figure 1:**
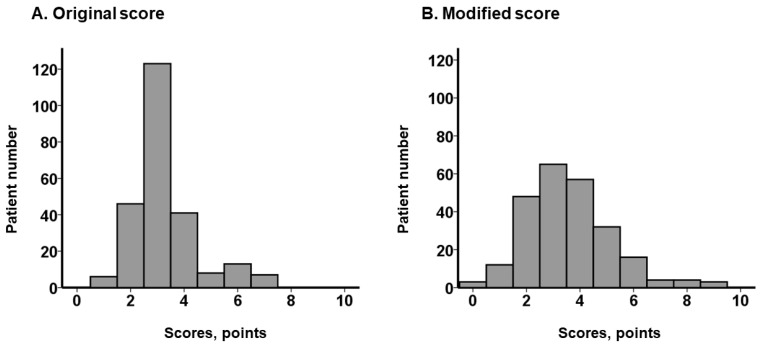
Distribution of the calculated H2FPEF scores: the original score (**A**) and the modified score (**B**).

H2FPEF score was calculated in all participants using baseline characteristics. Of note, almost no patients satisfied body mass index >30, and almost all participants satisfied age >60 years, both of which were major components of H2FPEF score. H2FPEF score was distributed relatively narrowly with a median value of 3 (3, 4) (Figure 1A). Examples of score calculation are displayed in the Appendix A. 

### 3.3. H2FPEF Score and Post-Procedural Clinical Outcome

All patients underwent successful TAVR. Patients were followed for a median of 730 (382, 730) days, with 730 days as a maximum observation duration. A total of 26 patients encountered the primary outcome defined as all-cause death and heart failure readmissions (12 death alone, 10 heart failure alone, and 4 for both). H2FPEF was significantly associated with the primary outcome with a hazard ratio of 1.33 (95% confidence interval 1.02–1.74, *p* = 0.036). 

### 3.4. Modified H2FPEF Score

Given the unique characteristics of TAVR candidates (elderly patients with advanced sarcopenia), the H2FPEF score was modified by updating the cutoffs of body mass index (from 30 to 23) and age (from 60 to 84), both of which were calculated using the receiver operating characteristics analyses for the primary outcomes. A modified H2FPEF score was calculated in all participants. The modified H2FPEF score was distributed widely with a median value of 3 (2, 4) (Figure 1B). Examples of the score calculation are displayed in Appendix A. 

### 3.5. Prognostic Impact of the Modified H2FPEF Score

**Figure 2:**
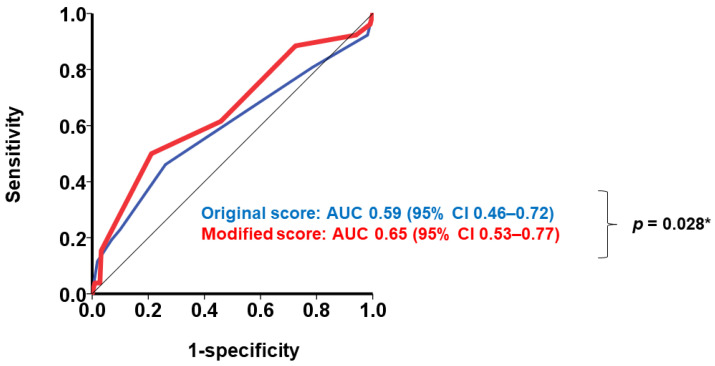
Receiver operating characteristics analyses of the original (blue line) vs. modified H2FPEF scores (red line) for predicting the primary outcome. The AUC was significantly higher in the modified H2FPEF score than the original H2FPEF score. AUC, area under the curve; CI, confidence interval. * *p* < 0.05.

The modified H2FPEF score was independently associated with the primary outcome with an adjusted hazard ratio of 1.22 (95% confidence interval 1.01–1.49, *p* = 0.047), which was adjusted for male sex and body mass index (Table 2). The predictability of the modified H2FPEF score, which was assessed using the area under the curve in the receiver operating characteristics analysis, was superior to the original H2FPEF score (0.69 vs. 0.59, *p* = 0.028; Figure 2). 

A modified H2FPEF score was not significantly associated with 2-year mortality with a hazard ratio of 1.17 (95% confidence interval 0.89–1.54, *p* = 0.25), whereas it was significantly associated with 2-year heart failure readmissions with a hazard ratio of 1.36 (95% confidence interval 1.05–1.77, *p* = 0.021). 

### 3.6. Stratification Using Modified H2FPEF Score

Patients were assigned to three groups according to their risk scores: a low-risk group (0–2 points, *n* = 63), an intermediate-risk group (3–5 points, *n* = 154), and a high-risk group (6–9 points, *n* = 27). The prevalence of patients who satisfied each item of the modified H2FPEF score was summarized in Table 3. 

**Figure 3:**
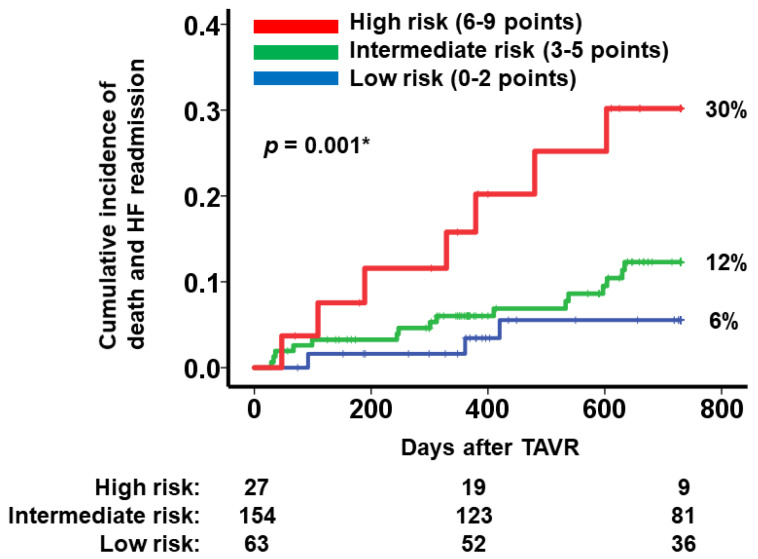
Cumulative incidence of the primary outcome during the 2-year observation period after TAVR stratified according to the modified H2FPEF score. Patients were stratified according to the modified H2FPEF score into three groups: high-risk group (score 6–9 points), intermediate-risk group (score 3–5 points), and low-risk group (score 0–2 points). * *p* < 0.05 via log-rank test.

The cumulative incidence of the primary outcomes during the 2-year observation period was significantly stratified into three risk groups (6%, 12%, and 30% for low-, intermediate-, and high-risk groups, *p* = 0.001; Figure 3). The hazard ratio of intermediate risk vs. low risk was 1.74 (95% confidence interval 0.63–4.85, *p* = 0.29). The hazard ratio of high risk vs. intermediate risk was 2.80 (95% confidence interval 1.15–6.81, *p* = 0.023). The sensitivity was 0.21, and the specificity was 0.89 in the high-risk group. The sensitivity was 0.93, and the specificity was 0.27 in the low-risk group. 

All 20 patients with left ventricular ejection fraction <40% were assigned to low- or intermediate-risk group, except for one patient. One patient at high risk had heart failure readmission on day 312. 

## 4. Discussion

In the present study, we evaluated the prognostic impact of the H2FPEF score [16], which was originally introduced to screen HFpEF patients, on the composite primary outcome consisting of all-cause death and heart failure readmissions during the 2-year observation period after TAVR. The original H2FPEF score was significantly associated with the 2-year primary outcome after TAVR. The modified H2FPEF score was constructed by updating the cutoffs of body mass index and age for better suitability to the current TAVR candidates (i.e., elderly patients with advanced sarcopenia). The predictability of the modified H2FPEF score for the primary outcome was superior to that of the original one. The cumulative incidence of the primary outcome was significantly stratified according to the modified H2FPEF score. 

### 4.1. HFpEF and H2FPEF Score

The accurate diagnosis of HFpEF is challenging [18]. The prevalence of HFpEF is increasing for several reasons, whereas HFpEF remains underdiagnosed so far. The gold standard for diagnosing HFpEF is a direct measurement of the elevated intra-cardiac pressure at rest or during exercise [19]. The H2FPEF score has been introduced as a convenient screening tool for HFpEF [16]. 

The utility of H2FPEF score to discriminate suspected HFpEF patients has been validated in various cohorts [20]. Furthermore, HFpEF score appears to be useful for the risk stratification of patients with various diseases, including HFpEF [17]. Given that patients with severe aortic stenosis have a similar pathology to HFpEF [13,14], we hypothesized that the H2FPEF score may also be useful for the risk-stratification of TAVR candidates. 

### 4.2. Prognostic Impact of H2FPEF Score

As hypothesized, the H2FPEF score had a prognostic impact on all-cause death and heart failure readmissions after TAVR. Several previous studies support our findings: The elevated intra-cardiac pressure, which was invasively measured after TAVR, was associated with worse clinical outcomes [21,22]. In another study, more advanced diastolic dysfunction, which was graded by echocardiography, was associated with a higher risk for 1-year mortality after TAVR than milder degrees of diastolic dysfunction [15]. Aortic stenosis itself can be treated with TAVR, but extra-valvular cardiac damage, including left ventricle, left atrium, mitral valve, pulmonary artery, and right ventricle, can persist even after TAVR [23]. Thus, it is reasonable that demographics and baseline hemodynamics items of the H2FPEF score, such as the presence of atrial fibrillation and E/e’ ratio, were associated with post-TAVR clinical outcomes. 

One previous study showed that H2FPEF score served as an independent predictor of adverse cardiovascular and heart failure outcomes after TAVR [11]. This study used the original H2FPEF score, whereas we modified the score to better fit current TAVR candidates: elderly patients with sarcopenia [24]. Almost no patients had a body mass index above 30, and almost all patients were aged over 60 years, both of which are cutoffs of the original H2FPEF score’s items. The modified H2FPEF score had greater predictability than the original one. We highly recommend to use the modified H2FPEF score in TAVR candidates, instead of the original one. 

### 4.3. Clinical Implication of the Modified H2FPEF Score

The H2FPEF score is convenient and can be calculated non-invasively using several simple parameters [16]. We further improved its predictability by modifying the cutoffs of several items: we reduced the cutoff of body mass index and increased the cutoff of age, because most TAVR candidates were elderly and had advanced sarcopenia. 

The score can be used for shared decision making before TAVR among clinicians, patients, and their relatives. Given the high specificity at the high risk score and the high sensitivity at the low risk score, we can expect/rule out the patients at risk of future adverse events. After TAVR, careful monitoring of worsening heart failure is highly recommended to prevent heart failure readmissions for the high-risk cohort. Post-TAVR prognosis may improve by intervening to several items of the H2FPEF score [25]. For example, cardiac rehabilitation may ameliorate the metabolism of visceral fat [26]. Catheter ablation for atrial fibrillation may improve atrial function [27]. Aggressive titration of heart failure medication may optimize post-TAVR hemodynamics [28].

### 4.4. Limitations

This study included a moderate-sized cohort from a single center. Given the small number of events, the included potential confounders in the multivariable analysis were limited. The profiles of HFpEF may vary depending on legions and ethics [29]. For example, few HFpEF patients in the Asian lesion have obesity. The applicability of our findings should be validated in larger multi-center studies including a variety of lesions and ethics. E/e’ ratio, one of the items of the H2FPEF score, may not necessarily be measured routinely in all institutes. We highly recommend to measure such echocardiographic data routinely before TAVR to calculate H2FPEF score. In this study, we preferred the H2FPEF score to the HFA-PEFF score. One of the limitations of the HFA-PEFF score is a requirement of more detailed echocardiographic data, including left atrial volume, left ventricular mass, and global strain. These may not necessarily be measured routinely before TAVR. Several variables in the H2FPEF score can be modifiable by any interventions. The prognostic impact of intervention on some of the items of H2FPEF score remains the next concern.

## 5. Conclusions

A modified H2FPEF score, which was originally constructed to diagnose the presence of HFpEF, could be used to risk-stratify elderly patients receiving TAVR. We constructed a modified H2FPEF score by updating the cutoffs of age and body mass index for better suitability to the current TAVR candidates (i.e., elderly patients with sarcopenia). The clinical utility of this score should be validated in future studies. 

## Figures and Tables

**Table 1:** Baseline characteristics.

	Total (*n* = 244)	High Risk (*n* = 27)	Low and Intermediate Risk (*n* = 217)	*p* Value
Demographics				
Age, years	86 (82, 88)	88 (86, 90)	85 (81, 88)	<0.001 *
Male sex	70 (29%)	10 (37%)	60 (28%)	0.21
Body mass index	21.5 (19.1, 24.3)	24.4 (21.8, 26.5)	21.1 (19.0, 23.9)	0.003 *
Systolic blood pressure, mmHg	114 (105, 125)	113 (103, 123)	114 (105, 126)	0.76
Pulse rate, bpm	71 (62, 77)	70 (61, 73)	71 (63, 78)	0.71
Comorbidity				
Hypertension	178 (73%)	24 (89%)	154 (71%)	0.039 *
Diabetes mellitus	38 (16%)	5 (19%)	33 (15%)	0.67
Dyslipidemia	116 (48%)	10 (37%)	106 (49%)	0.16
Atrial fibrillation	25 (10%)	17 (63%)	8 (4%)	<0.001 *
Coronary heart disease	63 (26%)	5 (19%)	58 (27%)	0.24
Peripheral artery disease	52 (21%)	8 (30%)	44 (20%)	0.2
History of heart failure admission	98 (40%)	17 (63%)	81 (37%)	0.011 *
History of cardiac surgery	12 (5%)	1 (4%)	11 (5%)	0.75
History of stroke	33 (14%)	6 (22%)	27 (12%)	0.18
Laboratory data				
Serum albumin, g/dL	3.8 (3.5, 4.0)	3.8 (3.5, 3.9)	3.8 (3.5, 4.0)	0.99
Hemoglobin, g/dL	11.4 (10.0, 12.5)	10.5 (10.0, 12.6)	11.4 (10.0, 12.5)	0.87
Serum sodium, mEq/L	140 (139, 142)	141 (138, 143)	140 (139, 142)	0.64
eGFR, mL/min/m^2^	49 (37, 62)	45 (29, 51)	50 (38, 64)	0.14
Plasma BNP, pg/mL	218 (118, 530)	184 (144, 495)	223 (114, 554)	0.85
Echocardiography				
LVDd, mm	46 (41, 51)	47 (44, 51)	45 (41, 50)	0.28
LVEF, %	64 (54, 70)	61 (53, 69)	65 (54, 70)	0.27
LVEF <40%	20 (8%)	1 (4%)	19 (8%)	0.37
Left atrial diameter, mm	43 (38, 50)	50 (45, 56)	43 (37, 48)	<0.001 *
Aortic valve parameter				
Peak velocity, m/s	4.4 (4.0, 4.9)	4.2 (3.8, 4.5)	4.5 (4.0, 4.9)	0.013 *
Mean pressure gradient, mmHg	46 (38, 57)	40 (34, 45)	47 (39, 59)	0.002 *
Valve area, cm^2^	0.6 (0.4, 0.7)	0.6 (0.5, 0.7)	0.5 (0.4, 0.7)	0.27
Moderate or greater MR	19 (8%)	5 (19%)	14 (6%)	0.027 *
Moderate or greater AR	22 (9%)	3 (11%)	19 (9%)	0.69
Moderate or greater TR	11 (5%)	5 (19%)	6 (3%)	<0.001 *
E/e’ ratio	16.3 (12.4, 23.1)	17.5 (12.5, 20.5)	16.1 (12.4, 23.3)	0.92
RVSP, mmHg	31 (26, 37)	39 (36, 43)	30 (25, 35)	<0.001 *
Medication				
Beta-blocker	74 (30%)	7 (26%)	67 (31%)	0.39
Renin-angiotensin system inhibitor	152 (62%)	16 (59%)	136 (63%)	0.44
Mineralocorticoid receptor antagonist	71 (29%)	7 (26%)	64 (29%)	0.45
Diuretics	133 (55%)	19 (70%)	114 (52%)	0.059
Scores				
STS score	5.2 (4.1, 7.4)	5.9 (4.5, 7.7)	5.2 (4.0, 7.4)	0.32
Modified H2FPEF score	3 (2, 4)	6 (6, 8)	3 (2, 4)	<0.001 *

Patients were stratified into three groups according to the modified H2FPEF score: low-, intermediate-, and high-risk groups. eGFR, estimated glomerular filtration rate; BNP, B-type natriuretic peptide; LVDd, left ventricular end-diastolic diameter; LVEF, left ventricular ejection fraction; MR, mitral regurgitation; AR, atrial regurgitation; TR, tricuspid regurgitation; RVSP, right ventricular systolic pressure. Continuous variable are stated as median and interquartile and compared between the two groups using Mann–Whitney U test. Categorical variables are stated as number and percentage and compared between the two groups using Fischer’s exact test. * *p* < 0.05.

**Table 2:** Potential predictors of the primary outcome including modified H2FPEF score.

	Univariable Analysis		Multivariable Analysis	
	Hazard Ratio (95% CI)	*p* Value	Hazard Ratio (95% CI)	*p* Value
Age, years	1.06 (0.97–1.15)	0.19		
Male sex	3.23 (1.49–7.14)	0.003 *	3.33 (1.51–7.14)	0.003 *
Body mass index	1.02 (1.00–1.03)	0.008 *	1.02 (1.00–1.03)	0.016 *
Serum albumin, g/dL	0.47 (0.20–1.12)	0.088		
eGFR, mL/min/1.73 m^2^	0.99 (0.97–1.01)	0.44		
Common logarithm of plasma BNP, pg/mL	1.58 (0.69–3.64)	0.28		
LVEF, %	1.01 (0.98–1.04)	0.58		
Heart failure history	1.89 (0.87–4.12)	0.11		
Atrial fibrillation	2.36 (0.89–6.25)	0.085		
Modified H2FPEF score, points	1.30 (1.06–1.58)	0.010 *	1.22 (1.01–1.49)	0.047 *

Potential predictors of the primary outcome (2-year death or heart failure readmission) were included in the univariable analyses. Variables with *p* < 0.05 in the univariable analyses were included in the multivariable analysis. eGFR, estimated glomerular filtration rate; BNP, B-type natriuretic peptide, LVEF, left ventricular ejection fraction. * *p* < 0.05 via Cox proportional hazard ratio regression analyses.

**Table 3:** Prevalence of patients who satisfied each item of modified H2FPEF score.

	High Risk (*n* = 27)	Low and Intermediate Risk (*n* = 217)	*p* Value
Bod mass index > 23	18 (67%)	72 (33%)	0.001 *
Hypertension	24 (89%)	154 (71%)	0.034 *
Atrial fibrillation	17 (63%)	8 (4%)	<0.001 *
RVSP > 35 mmHg	20 (74%)	49 (23%)	<0.001 *
Age > 84 years	26 (96%)	118 (54%)	<0.001 *
E/e’ ratio > 9	26 (96%)	202 (93%)	0.53

RVSP, right ventricular systolic pressure. Categorical variables are stated as number and percentage. * *p* < 0.05 via Fischer’s exact test.

## Data Availability

Data are available upon reasonable request from the corresponding author.

## Appendix A

Examples of score calculation:

A patient has the following profile: body mass index 24 (<30; 0 points), hypertension (1 point), atrial fibrillation (3 points), right ventricular systolic pressure 38 mmHg (>35 mmHg; 1 point), 70 years (>60 years; 1 point), and E/e’ ratio 10 (>9; 1 point). The H2FPEF score is calculated as 7 points. When we use the modified H2FPEF score, body mass index 24 (>23) is assigned a value of 2 points and age 70 years (<84 years) is assigned a value of 0 points. A total modified H2FPEF score is calculated as 8 points.

Another patient has the following profile: body mass index 21 (<23; 0 point), no hypertension (0 points), no atrial fibrillation (0 points), right ventricular systolic pressure 24 mmHg (<35 mmHg; 0 points), 87 years (>84 years; 1 point), and E/e’ ratio 5 (<9, 0 points). The modified H2FPEF score is calculated as 1 point.
